# Morphology of Ga_2_O_3_ Nanowires and Their Sensitivity to Volatile Organic Compounds

**DOI:** 10.3390/nano11020456

**Published:** 2021-02-11

**Authors:** Maciej Krawczyk, Patrycja Suchorska-Woźniak, Rafał Szukiewicz, Maciej Kuchowicz, Ryszard Korbutowicz, Helena Teterycz

**Affiliations:** 1 Faculty of Microsystem Electronics and Photonics, Wrocław University of Science and Technology, Wybrzeże Wyspiańskiego 27, 50-370 Wrocław, Poland; patrycja.suchorska-wozniak@pwr.edu.pl (P.S.-W.); ryszard.korbutowicz@pwr.edu.pl (R.K.); helena.teterycz@pwr.edu.pl (H.T.); 2 Institute of Experimental Physics, University of Wrocław, Maxa Borna 9, 50-204 Wrocław, Poland; rafal.szukiewicz@uwr.edu.pl (R.S.); maciej.kuchowicz@uwr.edu.pl (M.K.)

**Keywords:** chemical gas sensors, chemiresistors, nanomaterials, nanowires, gallium oxide, metal oxide, volatile organic compounds, acetone, ethanol, thermal synthesis

## Abstract

Gas sensitive structures made of nanowires exhibit extremally large specific surface area, and a great number of chemically active centres that can react with the ambient atmosphere. This makes the use of nanomaterials promising for super sensitive gas sensor applications. Monoclinic β-Ga_2_O_3_ nanowires (NWs) were synthesized from metallic gallium at atmospheric pressure in the presence of nitrogen and water vapor. The nanowires were grown directly on interdigitated gold electrodes screen printed on Al_2_O_3_ substrates, which constituted the gas sensor structure. The observations made with transmission electron microscope (TEM) have shown that the nanowires are monocrystalline and their diameters vary from 80 to 300 nm with the average value of approximately 170 nm. Au droplets were found to be anchored at the tips of the nanowires which may indicate that the nanowires followed the Vapor–Liquid–Solid (VLS) mechanism of growth. The conductivity of β-Ga_2_O_3_ NWs increases in the presence of volatile organic compounds (VOC) even in the temperature below 600 °C. The gas sensor based on the synthesized β-Ga_2_O_3_ NWs shows peak sensitivity to 100 ppm of ethanol of 75.1 at 760 °C, while peak sensitivity to 100 ppm of acetone is 27.5 at 690 °C.

## 1. Introduction

Growing demand for high sensitivity and selectivity of measurements in many branches of industry fosters intensive development of sensor technology. Among a wide variety of chemical gas sensors, the most commonly used are thick and thin film chemiresistors [1]. Parameters of chemiresistive gas sensors are determined by properties of gas receptor and transducer parts, sensor construction, and the type of detected gas [2,3]. The gas sensing layer of this type of sensors is typically made of semiconducting metal oxides SMOX (ZnO, SnO_2_, Ga_2_O_3_, WO_3_, Fe_2_O_3_, In_2_O_3_). It constitutes for the receptor part of the sensor, which has the most significant impact on the sensor selectivity and sensitivity [4,5]. Among many solutions used to improve the often unsatisfactory parameters of chemical gas sensors, the use of various forms of nanomaterials is especially worth mentioning.

The increasing interest in nanostructures of semiconducting metal oxides [6,7,8,9,10] comes from the fact that their radius is comparable with Debye length in a wide range of temperature and dopants concentration. As a result, electrical properties of nanomaterials strongly depend on the physicochemical processes that take place at their surface. Hence, gas sensors based on nanomaterials often exhibit a lower detection limit, higher sensitivity, and faster response and recovery times [11,12]. Belonging to the SMOX group, Ga_2_O_3_ is a promising gas sensing material, that can be synthesized at the nano scale.

β-Ga_2_O_3_ is a conducting, transparent metal oxide that belongs to a group of ultrawide bandgap materials (UWBG). Contrary to other UWBG materials, high quality bulk crystals of Ga_2_O_3_ are produced using melt-growth methods at relatively low cost. It is speculated that, in the nearest future, increased demand for gallium oxide substrates will result in further drop of their price to the level of sapphire substrates [13,14].

Gallium (III) oxide can crystalize in several polymorphic forms, with the most stable of them being β. The width of the bandgap is reported to slightly exceed 4.8 eV. The theoretical breakdown voltage of this material is higher than 8 MV/cm. The material itself is transparent to electromagnetic waves with a wavelength greater than 250 nm. Moreover, β-Ga_2_O_3_ is characterized by chemical and thermal stability. The thermal conductivity of this metal oxide is low and depends on the crystallographic direction [15,16,17].

Depending on the type and concentration of dopants, bulk crystals of β-Ga_2_O_3_ can be either isolators or *n*-type semiconductors. Although in the recent years a tremendous progress was made in synthesis of *p*-type epitaxial layers [18] and nanostructures [19] of β-Ga_2_O_3_, to our best knowledge, there are no reports in the literature of *p*-type doped bulk crystals of β-Ga_2_O_3_. Stoichiometric crystals of this metal oxide synthesized at high partial pressure of oxygen are isolators; therefore, for a long time *n*-type electrical conductivity of β-Ga_2_O_3_ was ascribed to the presence of oxygen vacancies in the bulk of the crystal [20]. However, more recent density functional theory simulations hint that the *n*-type conductivity of this material may result from unintentional doping with atoms of hydrogen or silicon (also Ge, Sn, F, Cl) [21,22]. Bulk crystals of β-Ga_2_O_3_ can be synthesized using various melt-growth methods, such as Czochralski, Bridgeman, and Edge-defined Film-fed Growth (EFG), also known as the Stepanov method. According to the literature [13,14,23], the diameters of monocrystalline β-Ga_2_O_3_ substrates with $\left(\bar{2}01\right)$ orientation, obtained with EFG method, reach up to 4”.

The gas sensing properties of this material were researched for the first time in the 1990s by M. Fleischer and H. Meixner [24]. They stated that conductivity of thin films of β-Ga_2_O_3_ depends on the concentration of oxygen in the ambient atmosphere. The change in conductivity of this material was ascribed to varying concentration of free carriers of electric charge due to adsorption and desorption of oxygen ions at its surface, and due to diffusion of oxygen to the bulk of the β-Ga_2_O_3_ crystal [20,25]. It was also stated that in the temperature above 500 °C, the resistance of thin and thick films of β-Ga_2_O_3_ changes in the presence of ethanol, methanol, carbon oxide, acetone and hydrogen [26,27]. Furthermore, Yu et al. [28] have noticed that even in the temperature as low as 100 °C, the conductance of a single β-Ga_2_O_3_ nanowire increases with the concentration of ethanol in the ambient atmosphere.

β-Ga_2_O_3_ in a form of one dimensional (1D) nanowires have a nearly circular cross section. Their diameter is usually in the range of single nanometers to hundreds of nanometers, and their length can be even several thousand times greater than the diameter. The specific surface area of nanowires is extremally large, and as a result there are many atoms at the surface that can react chemically with the ambient atmosphere. On this premise, it is widely acknowledged that the use of nanostructures, including nanowires and nanobelts, as gas sensing materials can greatly improve parameters of gas sensors [29]. However, reports in the literature on the use of β-Ga_2_O_3_ nanowires in VOC sensors are still rare. Jang et al. [30] have tested a response of Ga_2_O_3_ sensor to atmosphere containing 1000 ppm of acetone. They reported the peak sensitivity at 600 °C to be approximately 100. Park et al. [31] have reported sensitivity to 200 ppm of acetone and ethanol at 200 °C equal to approximately 1.5, and 2, respectively.

Among others, the following additive methods are used to synthesize β-Ga_2_O_3_ nanowires:

- thermal method [32];
- chemical vapor deposition (CVD) [33];
- metal-organic chemical vapor deposition (MOCVD) [34].

With these methods, large quantities of nanowires can be obtained at relatively low cost. It is generally assumed that the growth of metal oxide nanowires follows the Vapor–Liquid–Solid (VLS) or Vapor–Solid (VS) mechanisms, or a combination of both [35]. However, there are reports in the literature that the growth of β-Ga_2_O_3_ may follow a different mechanism. Sutter et al. [36] have synthesized β-Ga_2_O_3_ nanowires on SiO_2_ substrate with thermal method at 550–650 °C. They used Ga_2_S_3_ powder as the precursor, and Ar:H_2_ (2%) mixture as the carrier gas. These authors believe that under such conditions, the growth of β-Ga_2_O_3_ nanowires follows the VLS mechanism only in the initial phase of growth, during which a droplet of gold or silver metal determines the place of nucleation of the crystal. In the later phase, the nanowires follow a root growth mechanism. Kumar et al. [37] did not notice, characteristic to VLS mechanism, droplets of metal anchored at the tips of β-Ga_2_O_3_ nanowires synthesized with CVD method. However, they observed thickening at the base of the nanowires. On this basis, they propose the root growth as a probable mechanism.

Achieving nanowires of repeatable sizes is a major challenge of the aforementioned methods of synthesis. At high temperature of synthesis, metal droplets, that serve as a preferred place for nanowires growth, tend to creep along the substrate and agglomerate into larger clusters of atoms. This makes it much more difficult, if not impossible, to precisely control the place of nanowire nucleation. Due to the high temperature of synthesis, it is often necessary to use transitional substrates (carriers) from which nanowires should be detached and transferred to the target system. The transfer of the obtained nanostructures adds to the complexity of the entire process, and increases the difficulty of obtaining final structures with repeatable parameters.

This article presents the study of morphology of β-Ga_2_O_3_ nanowires grown directly on the gas sensor structure via thermal method at atmospheric pressure. Evidence for monocrystalline structure of nanowires synthesized in such conditions is shown with detailed analysis of results of transmission electron microscopy, X-ray photoelectron spectroscopy, and X-ray diffractometry. Moreover, contrary to previous reports in the literature, evidence for VLS mechanism of growth of β-Ga_2_O_3_ nanowires, such as metal particles at the top of the structures is presented. The presented study of sensitivity of β-Ga_2_O_3_ nanowires to VOC indicates that at elevated temperatures, the sensitivity of chemiresistive gas sensors based on this nanomaterial is greatly improved in comparison to, not only thin and thick films of Ga_2_O_3_, but also previous reports on β-Ga_2_O_3_ nanowires.

## 2. Materials and Methods

**Figure 1:**
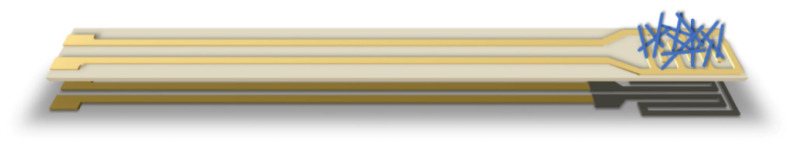
Al_2_O_3_ substrate with β-Ga_2_O_3_ nanowires grown on Au interdigitated electrodes. Pt meander-type heater was screen printed on the bottom side of the substrate.

The 25 mm × 2.5 mm × 0.63 mm alumina (96% Al_2_O_3_, CeramTec, Plochingen, Germany) substrates had been cleaned with hot isopropanol and then dried in a stream of dry nitrogen. Gold (8846-G, ESL Europe, Reading, UK) interdigitated electrodes (IDE) and leads had been screen printed on the top side of the alumina substrate and a platinum (5545, ESL Europe, Reading, UK) meander type resistor with gold (8846-G, ESL Europe, Reading, UK) leads, which served as a sensor heater, was screen printed on the bottom side of the substrate (Figure 1). Then the as-prepared substrate has been fired in a furnace at 850 °C for 10 min and let to cool down to room temperature. The area of IDE had been painted with a brush with a colloid water solution of (200 ppm) gold nanoparticles [38] and the substrates were dried at 50 °C.

Ga_2_O_3_ nanowires were grown on the as-prepared gold electrodes using a thermal method at atmospheric pressure. The nanowires were synthesized in a tubular resistance furnace in an atmosphere of humidified nitrogen. A flow of 0.9 L/min of nitrogen gas (Linde Gaz Polska Sp, z o.o., Krakow, Poland) was fed to the quartz tube reactor with a diameter of 50 mm. Before reaching the furnace, a portion of the flow (0.4 L/min) was directed to a humidifier filled with deionized (DI) water heated to 97 °C. The substrates and gallium metal (99.9999% purity, PPM Pure Metals GmbH, Langelsheim, Germany) were inserted in a specially built quartz boat. The surface of the interdigitated electrodes had been positioned approximately 5–6 mm above the droplet of gallium metal. The temperature of the furnace was raised and stabilized at 1012 °C, measured at the outer wall of the quartz tube with Pt-PtRh10 (Type S) thermocouple. Such conditions correspond to 1009 °C at the 20-centimeter-long growth zone inside the tube. With a swift move, the boat was then placed inside the hot quartz tube in which the temperature was maintained at 1012 °C (measured at the outer wall). The process of synthesis during which gallium metal was oxidizing and products of the reaction were thermally sublimating took approximately 60 min after which the boat was removed from the hot furnace and cooled at room temperature in ambient atmosphere.

The microstructure of the synthesized gallium oxide nanowires was observed with the scanning electron microscope (SEM) SU6600 (Hitachi Ltd., Hitachinaka, Japan) at 15 kV acceleration voltage, and at 200 kV acceleration voltage with the high-resolution transmission electron microscope (HRTEM) Tecnai G^2^ 20 X-TWIN (FEI, Hilsboro, OR, USA) equipped with LaB_6_ cathode, Eagle 2K CCD camera, and energy-dispersive spectroscopy (EDS) and scanning transmission electron microscopy (STEM) detectors.

The crystal structure of the synthesized gallium oxide nanowires was examined with the X-ray diffraction method using Materials Research Diffractometer XRD-MRD (Philips, Eindhoven, The Netherlands) with CuKα radiation. The average crystallite size and micro strain were determined with the Williamson-Hall method [39,40] based on the obtained diffractograms.

The surface chemical composition of the synthesized structures has been investigated with X-ray Photoelectron Spectroscopy (XPS). The non-monochromatized X-ray source based on Mg anode lamp (Mg Kα emission line) was used. High-resolution photoelectron energy spectra were recorded with AES/XPS system EA10 (Leybold-Heraeus GmbH, Cologne, Germany). Pressure in the chamber during the measurements was less than $1\times 10^{-9}$ mbar. All acquired spectra were calibrated to adventitious carbon C1s at 285 eV. The overall resolution of the spectrometer during the measurements was 0.96 eV as a full width of half maximum (FWHM) of the Ag3d5/2 line. CasaXPS software version 2.3 (Casa Software Ltd., Teignmouth, UK) was used for data deconvolution. The concentration of atoms in the sample was determined on the basis of XPS spectra analysis, taking into account the presence of individual elements O, C, Au and Ga.

**Figure 2:**
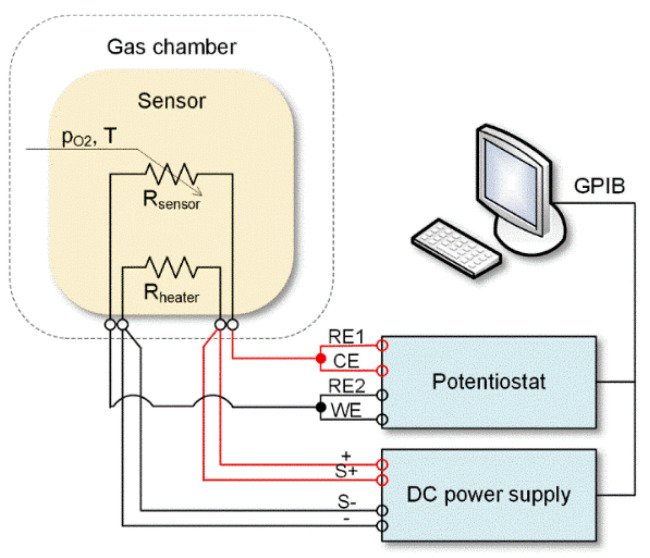
The temperature-controlled conductance measurement setup.

The electrical characterization of the obtained β-Ga_2_O_3_ nanowires was performed with the temperature stimulated conductance (TSC) method. During the measurements, the temperature of the Pt heater printed on the bottom of the substrate was changed linearly in the range of 300–900 °C at a rate of 2 deg/s. The heater supply voltage, generated by the DC power supply E3632A (Agilent Technologies Inc., Santa Clara, CA, USA), was controlled via a computer program with PID control loop (Figure 2). The gold interdigitated electrodes were polarized with 10 V_DC_ by the Potentiostat/Galvanostat 1287A (Solartron Analytical, Farnborough, UK) which simultaneously measured the current response of the synthesized nanowires with a sampling frequency of 1 Hz.

The substrate with the synthesized nanowires was kept inside a 5.5 L gas-tight glass chamber with a gas atmosphere of specified composition at 22 °C and relative humidity of approximately 30%. Acetone and ethanol were tested separately at concentrations of 100, 400, and 1000 ppm.

## 3. Results

### 3.1. Structural Characterization of β-Ga_2_O_3_ Nanowires

**Figure 3:**
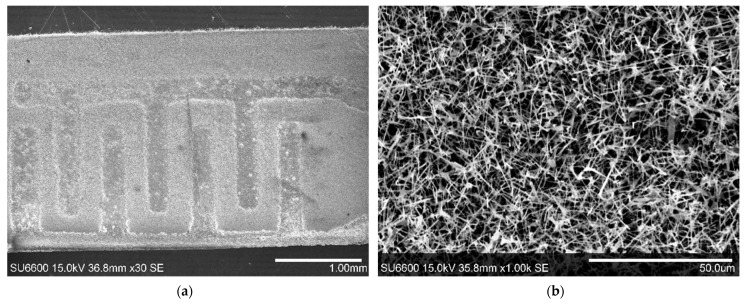
(**a**) SEM image of the top surface of the substrate with gold interdigitated electrodes and β-Ga_2_O_3_ nanowires; (**b**) ×1000 magnified image of β-Ga_2_O_3_ nanowires grown on the surface of Au electrode.

The observation of the top surface of the substrate with SEM has revealed that the distribution of the nanowires on the surface of the substrate is nonuniform (Figure 3a). The surface of the interdigitated gold electrodes is homogeneously covered by highly concentrated β-Ga_2_O_3_ nanowires (Figure 3b). Several nanowires with lengths in the range of hundreds of nanometers stick out over the edge of the substrate (Figure 3a). The examination with the SEM did not reveal any Ga-Au alloy droplets anchored at the tips of the nanowires, which are characteristic of the Vapor–Liquid–Solid mechanism of growth of nanowires.

**Figure 4:**
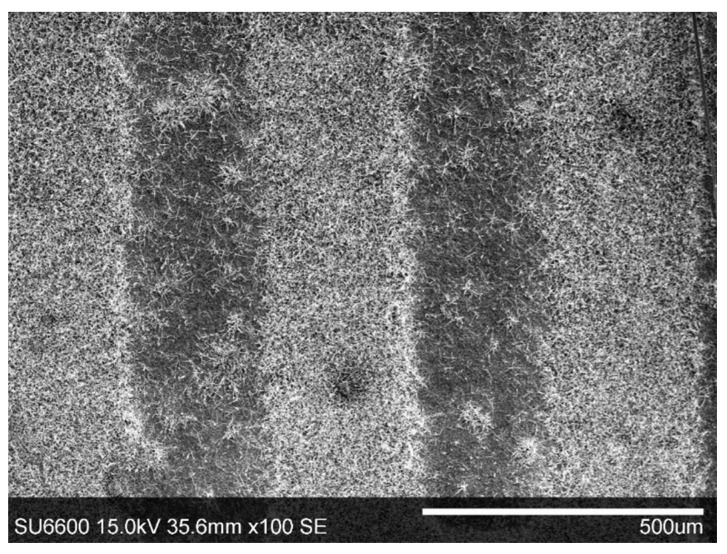
SEM image of the inter-electrode area/surface of the substrate with visible agglomerates formed by β-Ga_2_O_3_ nanowires.

However, it was noticed that the inter-electrode surface is sparsely populated by the β-Ga_2_O_3_ nanowires and their distribution is nonuniform. The nanowires form easily noticeable agglomerates (Figure 3a and Figure 4). We speculate that the preferred locations for the growth of the nanowires were the Au nanoparticles deposited from the water solution. Moreover, coagulation of the Au nanoparticles during drying of the solution, later resulted in the formation of the nanowire agglomerates.

**Figure 5:**
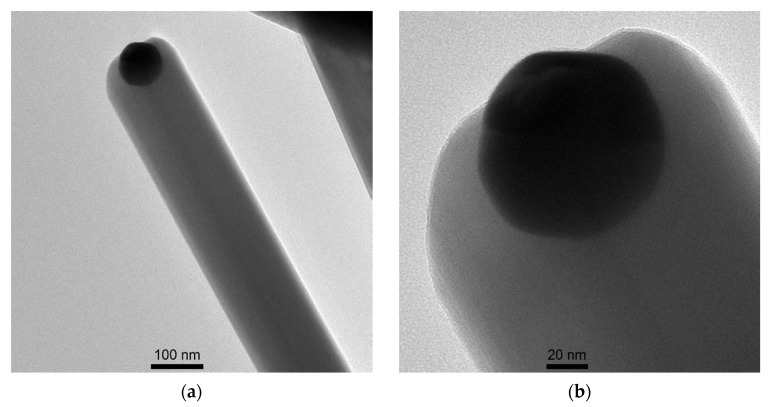
TEM image of (**a**) β-Ga_2_O_3_ nanowire; (**b**) Droplet of Au or Au-Ga alloy droplet at the top of the nanowire.

The synthesized nanowires had been separated from the substrate and their microstructure was examined with TEM. The observations have shown that the diameters of the β-Ga_2_O_3_ nanowires are nonuniform and vary from 80 to 300 nm. The average diameter is approximately 170 nm (Figure 5a). Moreover, TEM images have revealed droplets of Au or Au-Ga alloy anchored at the top of the nanowires with diameters roughly half of those of the nanowires (Figure 5b).

**Figure 6:**
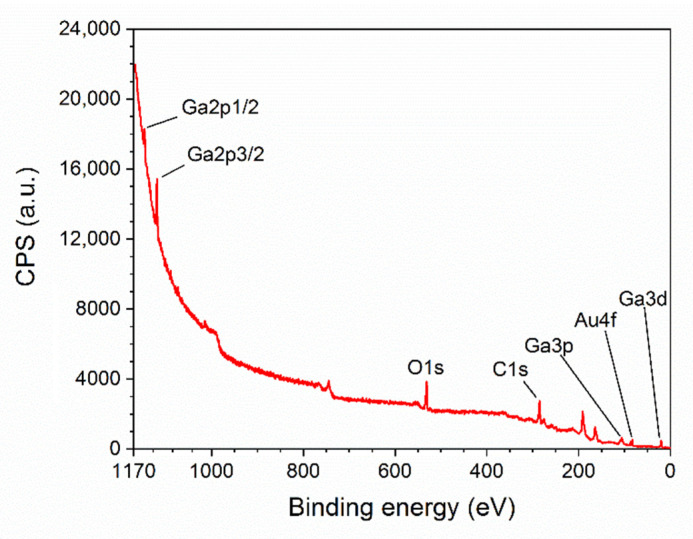
The chemical composition of β-Ga_2_O_3_ nanowire.

**Figure 7:**
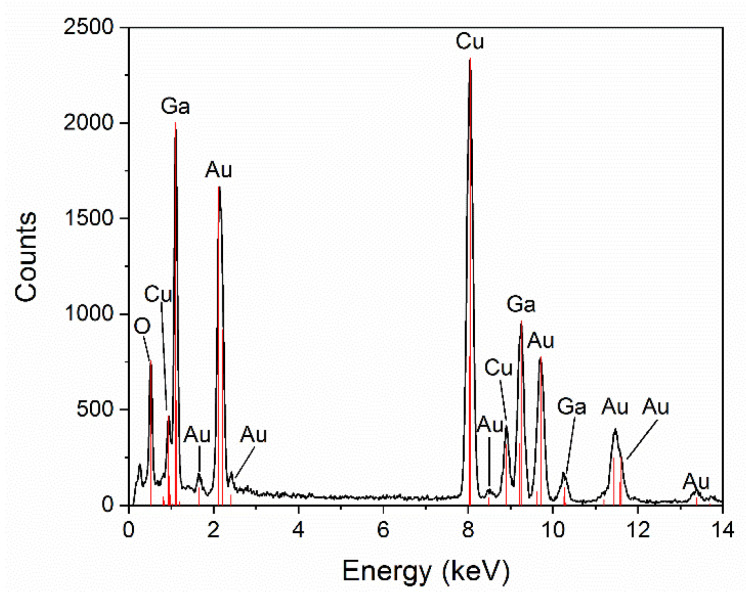
The chemical composition of Ga-Au alloy droplet anchored at the top of the nanowire.

The analysis of the chemical composition made with EDS detector installed in the high-resolution transmission electron microscope has shown that the nanowires are composed of pure gallium oxide (Figure 6), whereas the droplets present at the top of the nanowires, apart from gallium and oxygen, are composed of a large quantity of gold (Figure 7). The presence of Cu comes from the copper grid used during EDS measurements.

**Figure 8:**
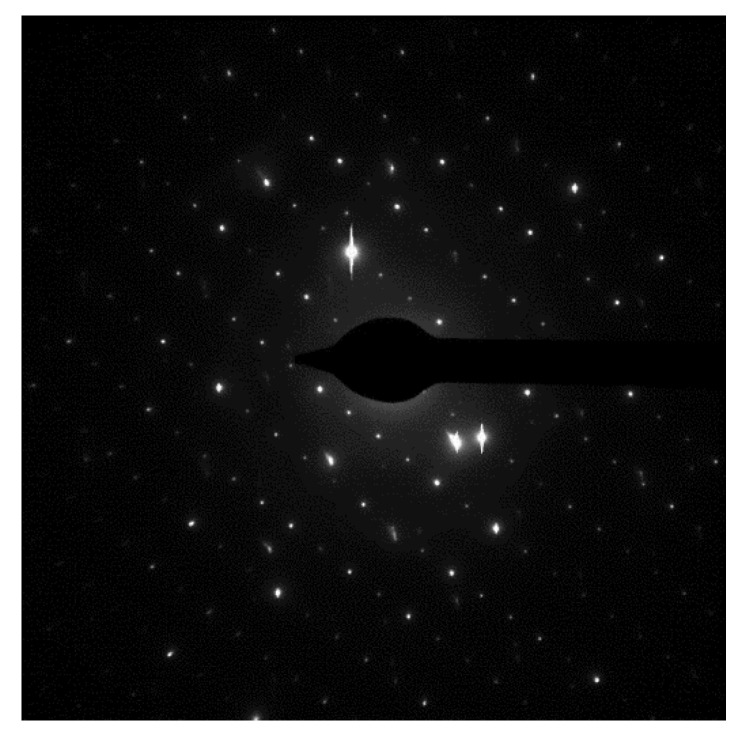
The diffraction pattern of an electron beam passing through β-Ga_2_O_3_ nanowire. The pattern reveals a monocrystalline structure of the nanowire.

The diffraction pattern of a beam of electrons passing through the examined sample points that the synthesized β-Ga_2_O_3_ nanowires are monocrystalline (Figure 8).

**Figure 9:**
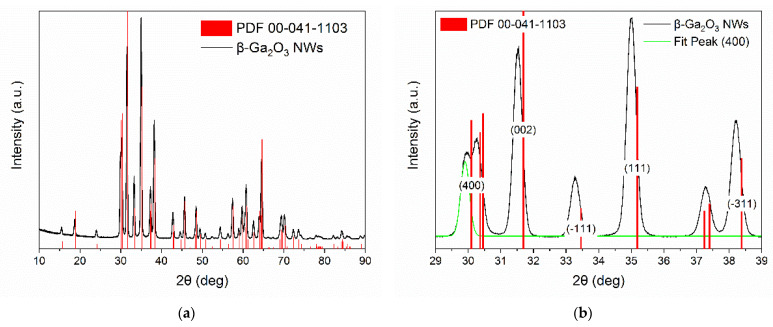
(**a**) X-ray diffractogram of β-Ga_2_O_3_ nanowires; (**b**) The most prominent peaks of the diffractogram in (**a**). All peaks in the diffractogram are shifted by, approximately, less than −0.20°.

The crystal structure of the nanowires synthesized on the surface of the alumina substrate and the gold electrodes has been identified using the X-ray diffraction (XRD) method. The analysis of the results has shown that gallium oxide grown on these surfaces is of monoclinic structure and belongs to space group C2/m (Figure 9). The most prominent peaks of the diffractogram correspond to the (002), ($\bar{1}$11), (111), and ($\bar{3}$11) crystallographic planes. Their corresponding angles are, accordingly, 31.52°, 33.28°, 35.00°, and 38.21°. Additionally, a peak corresponding to the (400) surface with a 2θ angle equal to 29.91° was fitted from the data (Figure 9b). All peaks visible in the diffractogram are shifted by, approximately, less than −0.20° relative to the peaks in a diffractogram of bulk β-Ga_2_O_3_ (PDF 00-041-1103). We speculate that this phenomenon could originate from the high shape factor of the nanowires [41].

Based on the obtained X-ray diffractogram, Bragg’s law (1), and the Equations (2)–(6), unit cell parameters were calculated as follows:

(1) nλ=2dsinθ ,

where: n=1—order of diffraction, λ=0.15406 nm—X-ray wavelength, d—interplanar spacing, θ—*a* scattering angle.

Then for a monoclinic system, the relationship between the interplanar spacing and the parameters of the unit cell is as follows:

(2) $$\frac{1}{d^{2}}=\frac{1}{\sin^{2}\beta }\left(\frac{h^{2}}{a^{2}}+\frac{k^{2}\sin^{2}\beta }{b^{2}}+\frac{\ell^{2}}{c^{2}}-\frac{2h\ell \cos\beta }{ac}\right)$$

where a, b, c, β—parameters of the unit cell; h, k, ℓ—Miller indices. By combining the Equations (1) and (2) we obtain

(3) $$\sin^{2}\theta =\frac{\lambda^{2}}{4\sin^{2}\beta }\left(\frac{h^{2}}{a^{2}}+\frac{k^{2}\sin^{2}\beta }{b^{2}}+\frac{\ell^{2}}{c^{2}}-\frac{2h\ell \cos\beta }{ac}\right)$$

and the unit cell parameters are

(4) $$a=\frac{2\lambda }{\sin\theta_{\left(400\right)}\sin\beta }$$

(5) $$b=\sqrt{\frac{\lambda^{2}}{4\left(\sin^{2}\theta_{\left(111\right)}-\frac{\lambda^{2}}{4\sin^{2}\beta }\left(\frac{1}{a^{2}}+\frac{1}{c^{2}}+\frac{2\cos\beta }{ac}\right)\right)}}$$

(6) $$c=\frac{2\lambda }{\sin\theta_{\left(002\right)}\sin\beta }$$

Solving the Equations (4)–(6) for β, the unit cell parameters have been determined to be: a=12.22 Å, b=2.939 Å, c=5.806 Å, and β=102.3° and are in an agreement with the literature data ascribed to β-Ga_2_O_3_ [16,42].

The micro strain and the average crystallite size of the nanowires have been determined using the Williamson–Hall method [39,40]. The broadening on peaks in diffractogram $\left(\beta_{T}\right)$ is caused by the strain $\left(\beta_{D}\right)$ and the crystallite size $\left(\beta_{\varepsilon }\right)$:

(7) $$\beta_{T}=\beta_{D}+\beta_{\varepsilon }=\frac{k\lambda }{D\cos\theta }+\frac{4\varepsilon \sin\theta }{\cos\theta }\;,$$

where: k—a shape factor (equal to 0.9 for a sphere), D—average crystallite size, ε—strain.

Transforming the Equation (7) to the following form—

(8) $$\beta_{T}\cos\theta =\varepsilon \left(4\sin\theta \right)+\frac{k\lambda }{D}\;,$$

—and plotting a linear function $\beta_{T}\cos\theta =f\left(4\sin\theta \right)$, the micro strain can be determined from the slope of the function and the average crystallite size can be determined from the y-intercept.

**Figure 10:**
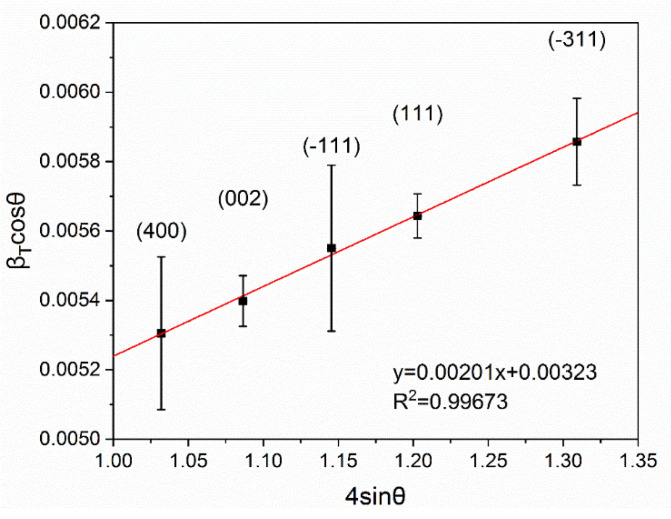
The Williamson–Hall plot of the β-Ga_2_O_3_ nanowires.

The five most prominent peaks in the X-ray diffractogram (Figure 9b) were used to calculate the strain and the average crystallite size. The micro strain has been determined from the Williamson–Hall plot (Figure 10) to be $\varepsilon =2.01\times 10^{-3}$ and the average crystallite size is D=42.39 nm (9):

(9) $$D=\frac{k\lambda }{C}\;,$$

where C—*y*-intercept.

The observations with SEM and TEM have shown that the length (≫10 μm) of the synthesized β-Ga_2_O_3_ nanowires is much larger than their diameter (~170 nm), therefore, based on [43] a shape correction factor α was incorporated to the Equation (9). The factor α is defined as a ratio of the surface area of a non-spherical particle (S’) to a surface area of a spherical particle (S) with their volumes being equal:

(10) $$\alpha =\frac{S’}{S}$$

Based on SEM and TEM images, we have estimated the shape of the nanowires to be, roughly, cylindrical with its length (25.5 μm) being 150 times its diameter (170 nm). Hence the radius of the nanowire is:

(11) $$h_{NW}=300r_{NW}$$

The volume of the nanowire and a spherical particle are equal—

(12) $$V_{NW}=\pi r_{NW}^{2}h_{NW}=300\pi r_{NW}^{3}=V_{sphere}=\frac{4}{3}\pi r_{sphere}^{3}\;,$$

—and so, the radius of the sphere is:

(13) $$r_{sphere}=4.22r_{NW}$$

The considered surface areas are as follows:

(14) $$S_{NW}=2\pi r_{NW}^{2}+2\pi r_{NW}h_{NW}=2\pi r_{NW}^{2}+2\pi r_{NW}\left(300r_{NW}\right)=301\left(2\pi r_{NW}^{2}\right)$$

(15) $$S_{sphere}=4\pi r_{sphere}^{2}=4\pi {\left(4.22r_{NW}\right)}^{2}=35.57\left(2\pi r_{NW}^{2}\right)\;,$$

therefore, the shape correction factor (10) is determined to be:

(16) $$\alpha =\frac{S_{NW}}{S_{sphere}}=4.07\;,$$

where $h_{NW},\;\;r_{NW},\;V_{NW},\;S_{NW}—$the length, radius, volume, and surface area of the nanowire approximated by a cylinder; $r_{sphere},\;\;V_{sphere},\;S_{sphere}—$the radius, volume, and surface area of the spherical particle.

Hence, the corrected average crystallite size is equal to:

(17) $$D=\frac{k\alpha \lambda }{C}=174.63\;\mathrm{nm}$$

The corrected value of the average crystallite size corresponds to the average diameter of the synthesized nanowires, although one has to keep in mind that the values determined by this method are only rough approximations [43]. However, the electron diffraction pattern indicates that the obtained nanostructures are nearly monocrystalline.

**Figure 11:**
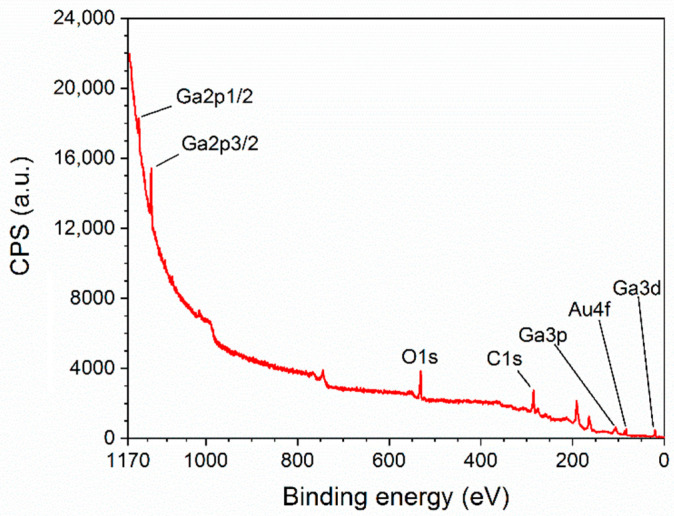
Survey spectrum of β-Ga_2_O_3_ nanowires.

The surface composition of β-Ga_2_O_3_ nanowires has been determined by X-ray Photoelectron Spectroscopy (XPS). The survey spectrum (Figure 11) consist of O (15.26%), C (58.46%), Au (0.9%), and Ga (25.38%) with specific atomic concentration in the brackets. The ratio of Ga/O is 0.6, instead of the theoretical value of 0.66. In this paper we omit the discussion of the carbon peak; however, core level spectra of C1s region consist mainly of C–C, C–O, and carbonates bonds. The origin of carbon is possibly related to environmental contamination and may also stem from the synthesis process of the nanowires.

**Figure 12:**
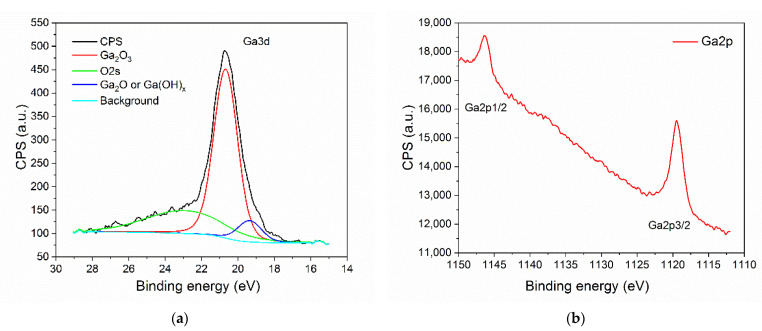
Core-level spectra of: (**a**) Ga3d; (**b**) Ga2p.

The analyzed sample consists mainly of Ga_2_O_3_ species which have been determined by deconvolution and analysis of Ga3d and Ga2p region (Figure 12). In the Ga3d region, the small peak visible at 19.5 eV may corresponds to either Ga_2_O or Ga-OH bond (Figure 12a). However, since the ratio of Ga/O has been determined to be 0.6, we believe that it is more likely that this peak corresponds to Ga-OH bond. The most prominent peak at energy of 20.6 eV relates to the presence of Ga_2_O_3_ at the surface [44,45]. O2s peak is present at the binding energy level of 23 eV. Region Ga2p also confirms the presence of Ga_2_O_3_ at the surface of the examined sample (Figure 12b). The Ga2p3/2 peak is visible at 1119.6 eV [44]. All observed peaks related to Ga are in agreement with literature data.

**Figure 13:**
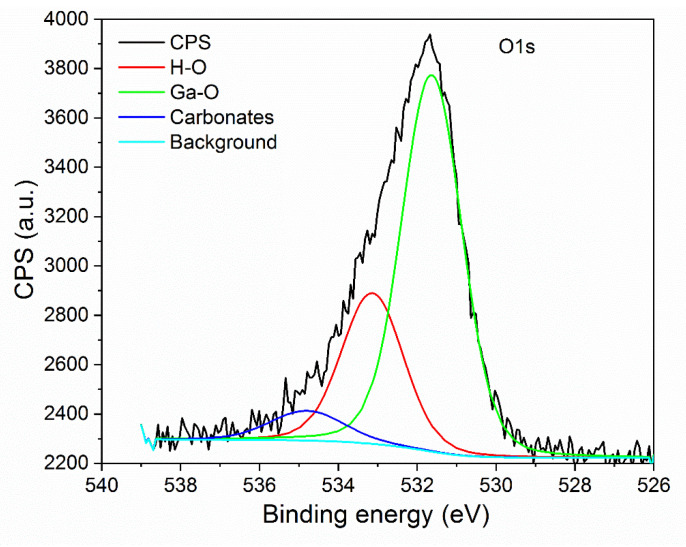
Core-level spectra of O1s region.

In the O1s region, the peak visible at binding energy of 531 eV is associated with network oxygen (Figure 13). The peak observed at 533.2 eV can be attributed to oxygen atoms of hydroxyl groups ${\mathrm{OH}}^{-}$. Carbonates visible in the spectrum may be a residue of the synthesis process.

**Figure 14:**
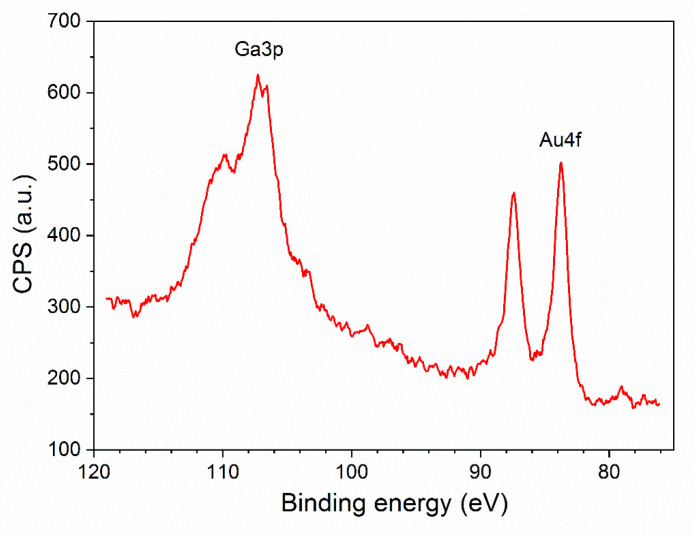
Core-level spectra of Au4f region.

Depending on the substrate material, the size of Au nanoparticles, and treatment conditions, the binding energy of the Au(4f) may be shifted [46,47]. However, in the examined sample, the peak Au4f visible at binding energy level of 84 eV is not shifted, and is characteristic for metallic Au (Figure 14).

### 3.2. Analysis of Gas Sensing Properties of β-Ga_2_O_3_ Nanowires

**Figure 15:**
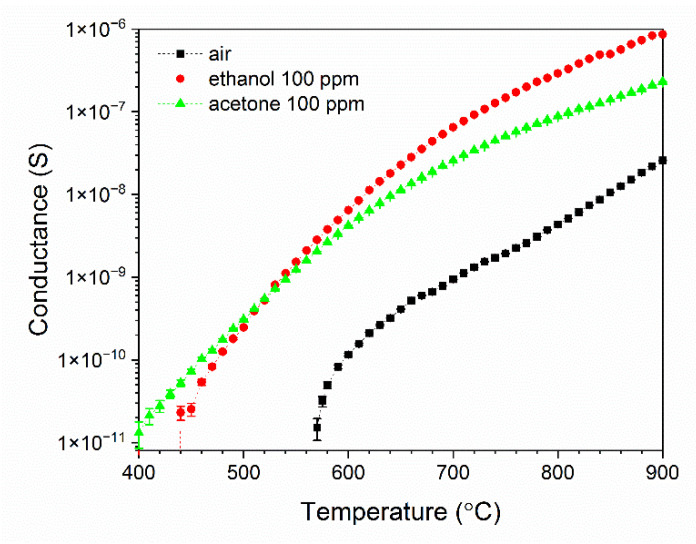
Change of conductance of β-Ga_2_O_3_ nanowires as a function of temperature in ambient air, and ethanol or acetone with the concentration of 100 ppm.

β-Ga_2_O_3_ nanowires have been characterized using temperature stimulated conductance method in which the electrodes were polarized with DC voltage while the temperature of Pt heater (Figure 1) was varied linearly and the current response of the system was measured. The analysis of the results has shown that the conductance of β-Ga_2_O_3_ nanowires is highly dependent on the temperature and the composition of the ambient atmosphere (Figure 15). The electrochemical interface device (1287A, Solartron Analytical, Farnborough, UK) used in the experiment allowed to measure the conductance in the range above 10^−11^ S which corresponds to the temperature above 400 °C. As the temperature increased from 400 °C to 900 °C, the conductance of β-Ga_2_O_3_ increased over several orders of magnitude. In ambient air with relative humidity of approximately 30%, the conductance rapidly increased above 565 °C (Figure 15).

The sensitivity of β-Ga_2_O_3_ nanowires to volatile organic compounds has been determined based on conductance measurements. This parameter is defined as a ratio of the conductance of the nanowires in an atmosphere containing the analyzed gas $G_{gas}$ to their conductance in ambient air $\left(G_{air}\right)$:

(18) $$S=\frac{G_{gas}}{G_{air}}$$

**Figure 16:**
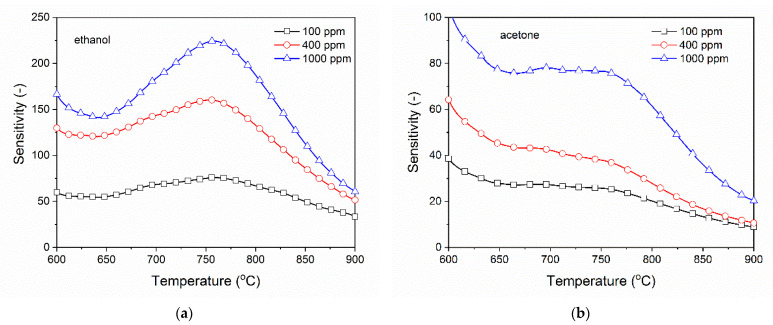
Sensitivity of β-Ga_2_O_3_ nanowires to volatile organic compounds: (**a**) The peak sensitivity to ethanol is at approximately 760 °C, (**b**) to acetone is at approximately 690 °C.

The sensitivity of β-Ga_2_O_3_ to organic volatile compounds is very high, especially to ethanol. The peak sensitivity to ethanol occurs at a temperature of around 760 °C (Figure 16a). The conductivity of the nanowires changes in a different manner when exposed to acetone. When vapors of this compound are present, the conductivity is increased in a broad range of temperatures between 650 °C and 760 °C and no particular peaks are visible (Figure 16b). This indicates that chemical processes occurring at the surface of gallium oxide are of a different character. In comparison to other metal oxide nanowires [48], β-Ga_2_O_3_ nanowires exhibit the maximum sensitivity at a much higher temperature, which is characteristic of gallium oxide gas sensing layers [30].

The resistivity of a porous polycrystalline metal oxide material ρ results from two components: resistivity of the grains boundaries $\rho_{gb}$ and resistivity of the grains $\rho_{g}$. For a fine-grain material the $\rho_{gb}$ component plays a major role:

(19) $$\rho_{gb}\left(T\right)=\rho_{g}\left(p_{O_{2}},T\right)\exp\left(\frac{eV_{s}}{kT}\right)\;,$$

where $\rho_{gb}$—resistivity of the grain boundaries, $\rho_{g}$—resistivity of the grains, $p_{O_{2}}$– oxygen partial pressure, $eV_{s}$—height of the energy gap between the grains.

Both of the factors in the Equation (19) depend on the oxygen partial pressure in the ambient and the temperature [2,49,50]. Whereas changes of the conductance result from physicochemical processes occurring at the surface of a gas-sensitive material and in its bulk (i.e., at the grains boundaries, at the grain contacts, in the near-electrode areas).

Depending on the microstructure of gas-sensitive materials, four pathways of electron conduction are considered [51,52]:

1. Surface conduction, when $r_{n}\gg L_{D}$;
2. At the grain boundary, when $r_{g}\geq L_{D}$;
3. In the volume of grains, when $r_{g}\leq L_{D}$;
4. At the Schottky contact;

where $r_{n}$—the radius of a connection between the grains (neck), $r_{g}$—the radius of the grain, $L_{D}$—width of the depleted layer (effective Debye length).

In the case of nanowires, three pathways are considered:

1. Surface conduction, when $r_{NW}\gg L_{D}$;
2. In the volume of the nanowire, when $r_{NW}\leq L_{D}$;
3. At the Schottky contact;

where $r_{NW}$—nanowire radius.

If the radius of the nanowire is larger than the effective Debye length, the surface plays an important role in the process of transport of an electric charge. Whereas if the radius of the nanowire is smaller than the Debye length, electrons from the volume become bound to oxygen chemisorbed at the surface, and then the conductance is determined by the volume of the nanowires [53,54].

So far, a universal model of the interaction of gases with the surface of semiconductor metal oxides has not been developed because their behavior is mainly determined by the type of gas-sensitive material, its physicochemical properties, the method of synthesis, dopants used, and the method of doping, material microstructure, and crystal structure. In the case of humid gas mixtures and organic compounds more complex than methane, the empirical relations are subject to very large errors. Most commonly, changes in sensor conductance at its working temperature are described by an experimental relation:

(20) $$G_{gas}\propto Ap_{gas}^{n}\;,$$

where A—a constant, n—an experimentally determined coefficient which is dependent on the working temperature, gas sensing material, material dopants used, detected gas. Because in this work, nanowires of undoped gallium oxide have been examined and the detected gases are volatile organic compounds very similar to each other, by combining Equations (18) and (20) we get:

(21) $$S=\frac{G_{gas}}{G_{air}}=\frac{Ap_{gas}^{n}}{G_{air}}=A’p_{gas}^{n}$$

where A’—a constant. Taking the logarithm of the Equation (21):

(22) $$\log S=\log A’+n\log p_{gas}$$

**Figure 17:**
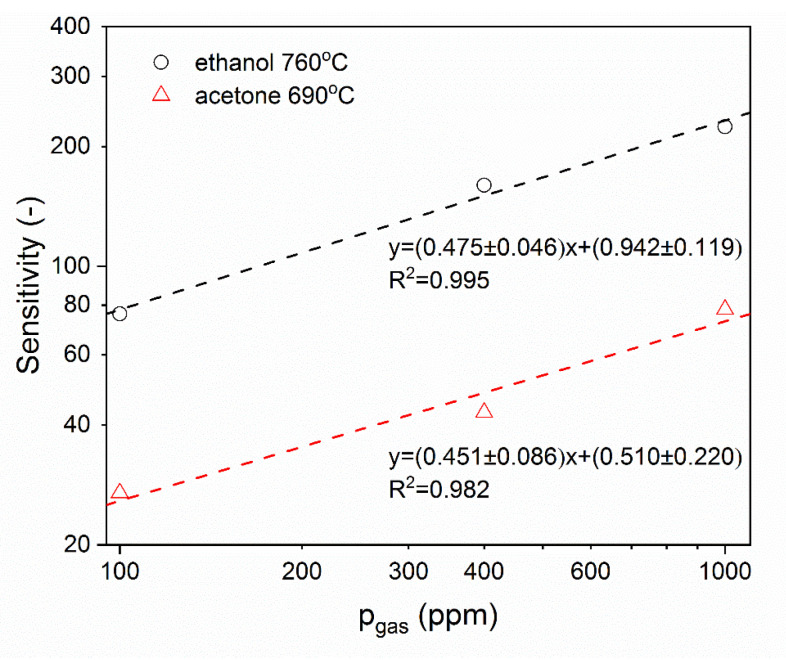
The sensitivity of β-Ga_2_O_3_ nanowires as a function of ethanol or acetone partial pressure.

By plotting, in a double-logarithmic system, the sensor sensitivity as a function of the partial pressure of the analyzed compound, the n coefficient has been determined (Figure 17).

In the case of ethanol, the n coefficient is equal to n=0.475±0.046, whereas in the case of acetone n=0.451±0.086. The determined values of the n coefficient are the same (within the margin of error) and are independent of the chemical structure of the detected volatile organic compounds. Since the n coefficient is independent of the composition of the detected atmosphere, it should mean that the chemical reactions with volatile organic compounds taking place on the surface of Ga_2_O_3_ proceed in multiple steps, but according to the same scheme [2].

According to [55], in the range of temperature in which peak sensitivity is observed, the examined volatile organic compounds become oxidized at material surface defects. The rate of oxidation v of the examined substances at the surface of the gas-sensing material is described by the following equation:

(23) $$v=k{\left[O^{-}\right]}^{a}p_{oc}^{n}\;,$$

where k—a constant (for a given reaction under specified conditions), $\left[O^{-}\right]$—concentration of oxygen ions chemisorbed at the surface of gas sensing material, $p_{oc}$—partial pressure of the detected gas; a, n—experimentally determined coefficients.

The concentration of chemisorbed oxygen ions depends on the concentration of active adsorption centers, which, in turn, is proportional to the active surface area:

(24) $$\left[O^{-}\right]\sim \left[\theta \right]\sim S_{SS}\;,$$

where [θ]—the concentration of active centers, $S_{SS}$—the active surface area.

With a larger surface area, and a greater number of active centers, and a higher concentration of chemisorbed oxygen ions, larger changes of the conductance are observed in the presence of vapors of volatile organic compounds. As the examined β-Ga_2_O_3_ nanowires exhibit extremely large specific surface, similarly to other nanostructures, their sensitivity towards ethanol and acetone vapors is very high.

However, large changes of the conductance of β-Ga_2_O_3_ in the presence of ethanol and acetone vapors could also result from the presence of gold particles in the examined metal oxide. As the observations made with HRTEM have shown, gold nanoparticles are anchored at the tips of the nanowires. They could influence the sensitivity of the metal oxide [56]. It can be assumed that Au atoms can occupy interstitial spaces in gallium oxide lattice increasing the sensitivity of the nanostructures. However, this is rather unlikely as the radius of Au atom is as high as 1.442 Å. As the analysis of the XPS results has shown, gold in the metallic form can be present at the surface of the nanowires, and, hence, influence their sensitivity. Further research aimed to verify this thesis is underway.

## 4. Conclusions

β-Ga_2_O_3_ nanowires were synthesized directly on interdigitated Au electrodes screen printed on Al_2_O_3_ substrate. The synthesis was carried using a thermal method at atmospheric pressure. The distribution of the nanowires in the inter-electrode area was not uniform, while the surface of the Au electrodes was densely and homogeneously populated by β-Ga_2_O_3_ nanowires. Therefore, future research will be focused on optimizing the process of synthesis of nanowires. Based on the observations made with HRTEM, a presence of gold or gold-gallium alloy was found, which hints that the nanowires have grown according to the Vapor–Liquid–Solid mechanism. Moreover, based on the analysis of HRTEM images, it has been found that the diameters of the nanowires vary from 80 to 300 nm. The average diameter is 170 nm, and is approximately equal to the average crystallite size, which is determined based on the analysis of XRD results. Therefore, it should be stated that the individual nanowires are monocrystalline. The electrical characterization has shown that the conductance of β-Ga_2_O_3_ nanowires is clearly dependent on the temperature and the composition of the ambient atmosphere. The conductivity of these nanostructures increases very strongly in the presence of vapors of volatile organic compounds, namely, ethanol and acetone. These changes may be due to the very large active surface area of the nanowires and the presence of gold nanoparticles at the surface and/or in the volume of the gallium oxide. Further works will be dedicated to the optimization of the process of nanowire synthesis and investigation of the influence of gold on the sensitivity of β-Ga_2_O_3_ nanowires towards vapors of volatile organic compounds.

## Data Availability

No new data were created or analyzed in this study. Data sharing is not applicable to this article.
